# Coil Embolization of Coronary-Cameral Fistula Complicating Revascularization of Chronic Total Occlusion

**DOI:** 10.1155/2018/6857318

**Published:** 2018-08-29

**Authors:** Amy Mertens, Pratik Dalal, Michael Ashbrook, Ivan Hanson

**Affiliations:** ^1^Department of Cardiovascular Medicine, Beaumont Health System, Royal Oak, MI, USA; ^2^Oakland University William Beaumont School of Medicine, Royal Oak, MI, USA

## Abstract

Traumatic vessel perforation is a potential complication of chronic total occlusion (CTO) percutaneous coronary artery intervention (PCI). A rare consequence of this complication is a coronary-cameral fistula. The management of this condition is not well elucidated. Herein, we present such a case of symptomatic left anterior descending to the right ventricle (LAD-RV) fistula which was treated with coil embolization.

## 1. Introduction

Coronary artery chronic total occlusion (CTO) is present in 18–26% of all patients with coronary disease and in up to 50% of patients after coronary artery bypass grafting [1, 2]. These procedures are known to have higher complication rates compared to traditional PCI and require careful procedural planning and case selection for success. Potential complications include coronary perforation with or without tamponade due to equipment exit or stent overdilation, vessel thrombosis or occlusion, high radiation exposure leading to skin injury, vascular access complications, and contrast nephropathy [3]. Iatrogenic coronary artery fistulas are seen in only 0.25% of cases and typically involve coronary artery to coronary vein communication [4, 5]. We present a case of a left anterior descending (LAD) artery to the right ventricle (RV) fistula complicating a LAD CTO revascularization.

## 2. Case Presentation

**Figure 1:**
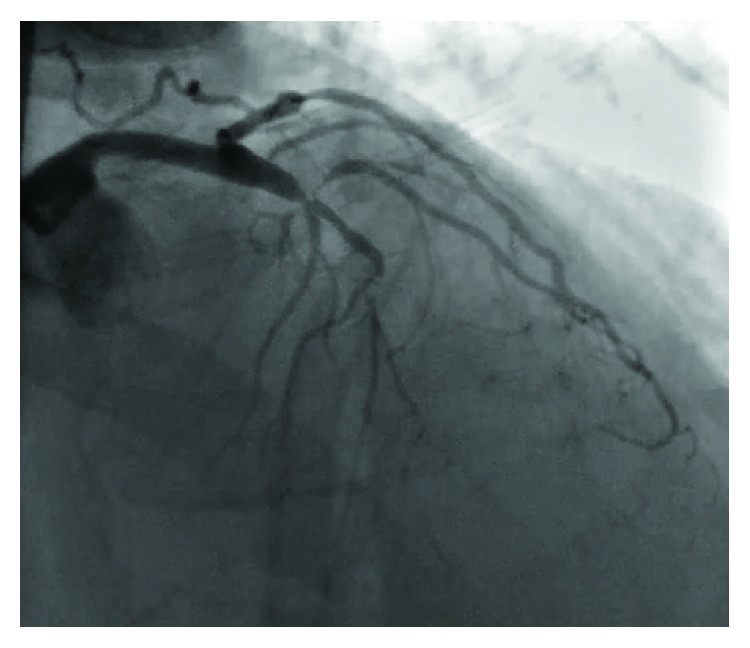
Left coronary angiography demonstrating severe stenosis of the left circumflex artery and CTO of the LAD.

**Figure 2:**
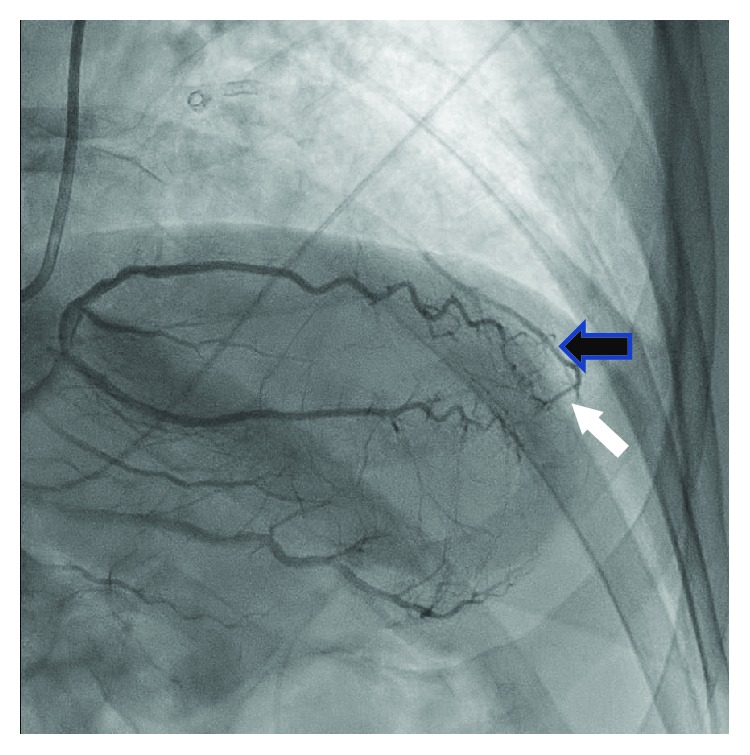
Epicardial vessel from the distal right coronary artery (white arrow) collateralizing the diagonal branch (black arrow).

**Figure 3:**
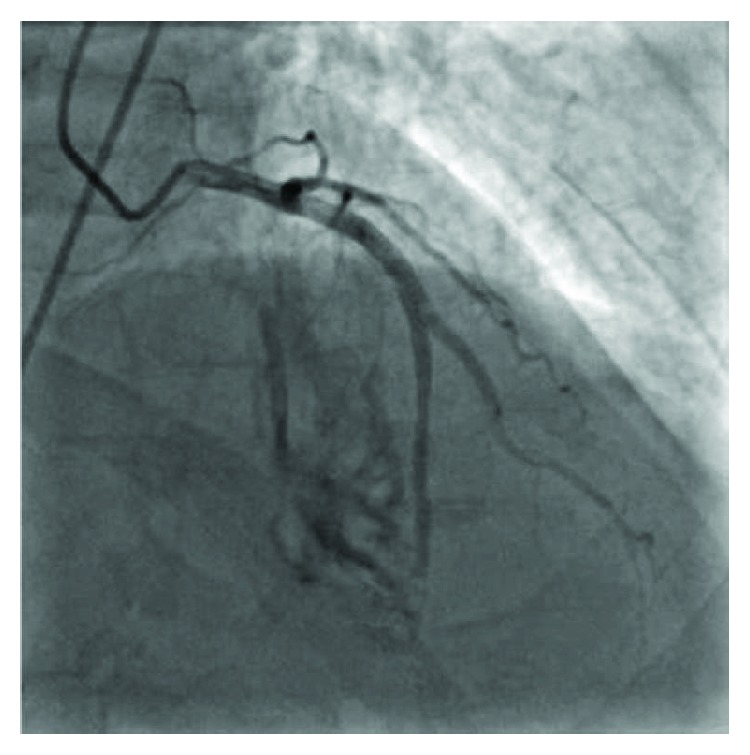
Angiography of the LAD demonstrating an iatrogenic LAD to RV fistula.

A 71-year-old man with progressive angina was found to have severe stenosis in the circumflex artery and complex CTO of the left anterior descending artery (Figure 1, Supplementary Video 1). Coronary artery bypass surgery was offered to the patient but declined. He underwent uncomplicated stenting of the circumflex artery, followed by staged CTO recanalization of the LAD. A guidewire was passed relatively easily across the occlusion into the true lumen of the distal LAD beyond the bifurcation. Antegrade wiring of the diagonal artery was difficult. An epicardial collateral from the distal right coronary artery was used to access the diagonal branch retrograde, and this wire was steered into the antegrade guide catheter and externalized (Figure 2). Both the LAD and diagonal were dilated with 3.0 mm noncompliant balloons. The diagonal was stented into the proximal LAD, and the distal LAD was rewired. The distal LAD was dilated through the stent struts to allow passage of stents into the distal LAD (Culotte technique). The distal LAD was stented using four everolimus drug-eluting stents. The stents were postdilated with 3.0 mm noncompliant balloons in the diagonal and distal LAD and 4.0 mm noncompliant balloon in the proximal LAD. Within the distal-most stent in the distal LAD, the balloon had a persistent waist until an inflation pressure of 12 ATM. At that point, the balloon suddenly expanded. Angiography revealed contrast filling of the right ventricle (RV), with the appearance of one or two focal jets of contrast extravasation at the location of the rigid lesion in the distal LAD, consistent with iatrogenic LAD-RV fistula (Figure 3, Supplementary Video 2). Of note, the flow beyond the stents in the distal LAD was not seen, likely due to shunt flow and “coronary steal.” The patient remained hemodynamically stable and was asymptomatic, and it was elected to manage the fistula conservatively. Serial echocardiograms revealed only trace pericardial effusion. He was discharged in stable condition.

Approximately three weeks after the CTO procedure, the patient was presented to the emergency department with chest pain and dyspnea. He described several episodes of “tearing” sensation in the chest. Blood pressure and heart rate were 123/69 mmHg and 64 beats per minute, respectively. A 12-lead ECG revealed inferior T wave inversion. Troponin I was elevated to 0.06 ng/mL. Urgent coronary angiography was performed, which revealed patent stents in the proximal LAD and diagonal branch. LAD-RV fistula appeared relatively unchanged compared to during the CTO procedure (Figure 3). The right coronary artery was normal and provided a very faint collateral to the apical LAD. Left ventriculography in the left anterior oblique projection revealed no evidence of a ventricular septal defect. The patient developed profound hypotension of unclear etiology during angiography. A right heart catheterization revealed normal filling pressures, normal cardiac output and ratio of pulmonic to systemic flow (Qp:Qs) 1.7. A transthoracic echocardiogram revealed preserved ejection fraction and normal left ventricular wall motion with turbulent flow signals at the LV and RV apex throughout the cardiac cycle (Supplementary Video 3). There was no pericardial effusion. The patient was transferred to the cardiac intensive care unit in stable condition.

Heart team evaluation was undertaken, and it was decided to perform coil embolization of the distal LAD. A standard 6 French left coronary guide catheter was used to advance a ProGreat (Terumo Medical Corp., Somerset, New Jersey) guidewire and 2.8 French catheter into the distal LAD. This was used to deploy two Ruby (Penumbra, Inc., Alameda, California) coils at the distal end of the stent.

**Figure 4:**
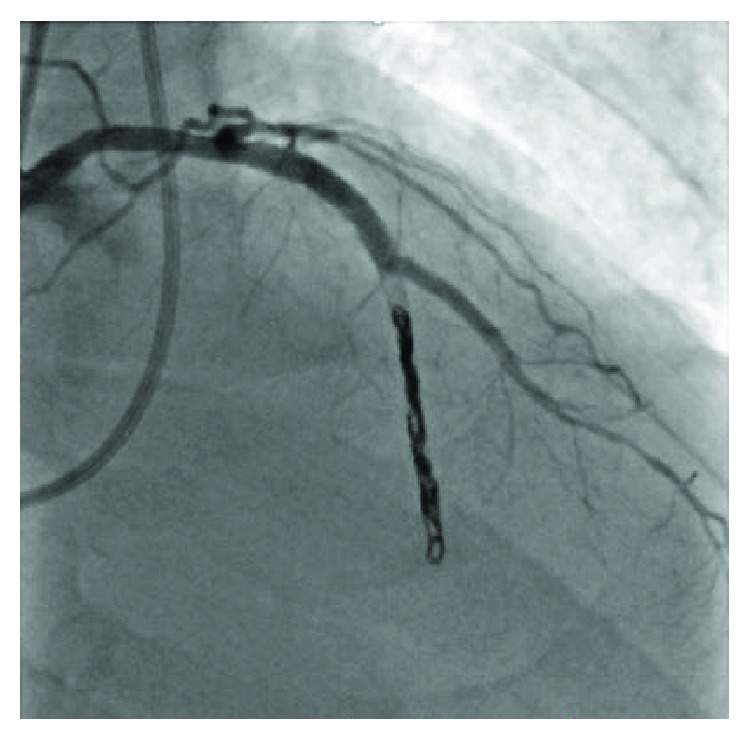
Angiography of the LAD showing successful coil embolization and complete cessation of flow into the RV.

There was still persistent shunt flow, so two Tornado (Cook Medical, Bloomington, Indiana) coils were subsequently deployed. Angiogram of the LAD confirmed complete cessation of flow into the distal LAD and absence of shunt flow (Figure 4, Supplementary Video 4). Hemodynamics postprocedure demonstrated a 20 mmHg increase in systolic blood pressure and normalization of Qp:Qs. A follow-up echocardiogram revealed the obliteration of apical shunt flow, normal left ventricular ejection fraction, and no left ventricular wall motion abnormalities. Creatine kinase eight-hour postprocedure was normal. The patient was discharged home in stable condition. At follow-up, one month later, the patient remained asymptomatic.

## 3. Discussion

To our knowledge, this is the first reported case of a traumatic LAD to RV fistula precipitated by CTO PCI that was successfully closed with percutaneous coil embolization. There have been 3 other reported cases of iatrogenic coronary artery fistulae in the literature, two of which were caused by RCA guidewire perforation and one secondary to LAD stent deployment [6, 7].

Development of LAD-RV fistula in this patient may have related to stent/balloon oversizing and rupture of the relatively small distal vessel. It is conceivable that the distal LAD had an intramyocardial course, which could explain why flow was preferentially tracked into RV, and not the pericardial space. The symptoms of chest pain, described as “tearing,” along with low-level troponin release, may have been caused by the ongoing myocardial rupture. Although there was a significant shunting, there was no evidence of typical angina, which may have been expected if coronary steal had occurred [8].

Definitive management of symptomatic coronary-cameral fistula has not been well elucidated. Approaches may include conservative management, surgical ligation, prolonged balloon inflation, covered stents, and device embolization [9, 10].

The GRAFTMASTER (Abbott Vascular, Santa Clara, California) is a polytetrafluoroethylene- (PTFE-) covered stent that can be delivered into the coronary arteries via a 6 French guide catheter. The crimped profile of the device is 1.63–1.73 mm, making it difficult to deliver in tortuous or calcified/stented vessels. The smallest expanded stent diameter is 2.8 mm, so for smaller vessels, it is less useful. In addition, the GRAFTMASTER requires guidewire “purchase” beyond the site of deployment, but in the present case, distal intraluminal guidewire position could not be confirmed. Deployment of a covered stent has a potential advantage of maintaining distal flow in the vessel beyond the treatment site. If covered stent deployment is not possible, device embolization can be considered.

Coil embolization requires placement of a delivery catheter to the segment of interest, followed by advancement of coils, which are initially elongated and then conformed to their prespecified shape and volume once they are extruded. Ruby is a detachable coil, so it can be withdrawn or completely removed if needed. This type of coil was selected for initial delivery in the present case in order to achieve a precise position and avoid extension of the coil into the fistula tract or RV. Tornado is available in thickness as small as 0.018″ and is coated with thrombogenic fronds to promote faster hemostasis. It is not detachable.

In the present case, distal LAD embolization resulted in complete occlusion of the stented segment beyond the large diagonal branch. It is important to note that embolization may not have been the preferred strategy if the perforation had occurred in a larger vessel or one supplying a large myocardial territory. The lack of a rise in cardiac biomarkers and the absence of left ventricular wall motion abnormalities suggested that the apical LAD and interventricular septum were adequately collateralized. Finally, the large diagonal branch, which was felt to be a more important vessel due to its large size and myocardial territory, was preserved.

## 4. Conclusion

Traumatic vessel perforation leading to LAD-RV fistula is a potential complication of CTO PCI and may lead to untoward symptoms and significant intracardiac shunting. A working knowledge of transcatheter closure techniques, including covered stents and device embolization, is mandatory for coronary interventionalists and CTO operators in particular.

## Supplementary Materials

Supplementary Materials Supplementary Video 1: Left coronary angiography demonstrating severe stenosis of the left circumflex artery and CTO of the LAD. Supplementary Video 2: Angiography of the LAD demonstrating an iatrogenic LAD to RV fistula. Supplementary Video 3: 2D echocardiography displaying a turbulent color flow signals at the apex of the left and right ventricle. Supplementary Video 4: Angiography of the LAD showing successful coil embolization and complete cessation of flow into the RV.
