# Supplementary material for: Coil Embolization of Coronary-Cameral Fistula Complicating Revascularization of Chronic Total Occlusion
Source: Case Rep Cardiol. 2018 Aug 29;2018:6857318. doi: 10.1155/2018/6857318 (PMC6136583; doi:10.1155/2018/6857318)
Supplement: Supplementary Materials — Supplementary Video 1: Left coronary angiography demonstrating severe stenosis of the left circumflex artery and CTO of the LAD. Supplementary Video 2: Angiography of the LAD demonstrating an iatrogenic LAD to RV fistula. Supplementary Video 3: 2D echocardiography displaying a turbulent color flow signals at the apex of the left and right ventricle. Supplementary Video 4: Angiography of the LAD showing successful coil embolization and complete cessation of flow into the RV. [file 6857318.f1.zip › Supplementary Videos/Supplementary Materials.docx]

**Supplementary Materials**

Supplementary Video 1: Left coronary angiography demonstrating severe stenosis of the left circumflex artery and CTO of the LAD.

Supplementary Video 2: Angiography of the LAD demonstrating an iatrogenic LAD to RV fistula.

Supplementary Video 3: 2D echocardiography displaying a turbulent color flow signals at the apex of the left and right ventricle.

Supplementary Video 4: Angiography of the LAD showing successful coil embolization and complete cessation of flow into the RV.
